# circ_0002346 Suppresses Non-Small-Cell Lung Cancer Progression Depending on the Regulation of the miR-582-3p/STXBP6 Axis

**DOI:** 10.1155/2021/1565660

**Published:** 2021-10-20

**Authors:** Weijie Wang, Yi Lin, Guanghui Zhang, Guofu Shi, Yongsheng Jiang, Wentao Hu, Wei Zuo

**Affiliations:** ^1^Department of Thoracic Surgery, The Affiliated Xiangshan Hospital of Wenzhou Medical University, Ningbo, Zhejiang 315000, China; ^2^Department of Respiration, The Affiliated Xiangshan Hospital of Wenzhou Medical University, Ningbo, Zhejiang 315000, China; ^3^Science and Education Management Center, The Affiliated Xiangshan Hospital of Wenzhou Medical University, Ningbo, Zhejiang 315000, China; ^4^Department of Thoracic Surgery, Ningbo First Hospital, Ningbo, Zhejiang 315000, China; ^5^Department of General Surgery, The Affiliated Xiangshan Hospital of Wenzhou Medical University, Ningbo, Zhejiang 315000, China

## Abstract

**Background:**

Accumulating articles have reported the pivotal regulatory roles of circular RNAs (circRNAs) in non-small-cell lung cancer (NSCLC) tumorigenesis. Here, our purpose was to explore the role of circ_0002346 in NSCLC progression and its associated mechanism.

**Methods:**

Cell proliferation ability was assessed by a 5-ethynyl-2′-deoxyuridine (EDU) assay and a colony formation assay. Transwell assays were conducted to analyze cell migration and invasion abilities. Cell apoptosis was analyzed by flow cytometry and by using a caspase3 activity assay kit. The glycolysis of NSCLC cells was analyzed using a fluorescence-based glucose/lactate assay kit. A dual-luciferase reporter assay and an RNA pull-down assay were performed to verify the binding relationship between microRNA-582-3p (miR-582-3p) and circ_0002346 or syntaxin-binding protein 6 (STXBP6).

**Results:**

circ_0002346 level was prominently downregulated in NSCLC tissues and cell lines. circ_0002346 overexpression significantly suppressed the proliferation, migration, invasion, and glycolysis and triggered the apoptosis of NSCLC cells. circ_0002346 directly interacted with miR-582-3p, and circ_0002346 overexpression-mediated antitumor effects in NSCLC cells were partly reversed by miR-582-3p overexpression. miR-582-3p directly interacted with the 3′ untranslated region (3′UTR) of STXBP6, and STXBP6 silencing partly counteracted circ_0002346 overexpression-mediated antitumor influences in NSCLC cells. circ_0002346 can upregulate the expression of STXBP6 by acting as a miR-582-3p sponge in NSCLC cells. circ_0002346 overexpression suppressed xenograft tumor growth *in vivo*.

**Conclusion:**

circ_0002346 overexpression suppressed the malignant properties of NSCLC cells by binding to miR-582-3p to induce the expression of STXBP6.

## 1. Introduction

Non-small-cell lung cancer (NSCLC) is a predominant type of lung cancer, including adenocarcinoma, squamous cell carcinoma, and large cell carcinoma [1, 2]. Due to the lack of effective markers for early diagnosis, most patients are diagnosed with NSCLC at an advanced stage, leading to poor prognosis [3, 4]. Therefore, the development of novel and effective diagnostic, therapeutic, and prognostic markers is important in the treatment of NSCLC.

Circular RNAs (circRNAs) are natural noncoding RNAs characterized by a closed circular structure [5, 6]. circRNAs are conserved during evolution, with high stability and a tissue-specific expression pattern. These characteristics make circRNAs ideal markers for human diseases [7, 8]. circRNAs are widely dysregulated in NSCLC, and accumulating evidence has identified a series of circRNA biomarkers for NSCLC, including circ_0001649 [9], circ_0043278 [10], and circ_0102533 [11]. We investigated the function of a novel circRNA, circ_0002346, in NSCLC progression.

MicroRNAs (miRNAs), as another class of noncoding RNAs, play important regulatory roles in NSCLC progression as oncogenes or tumor suppressor genes [12, 13]. circRNAs can indirectly regulate gene expression by sequestering miRNAs, thus releasing the downstream genes from the inhibition of miRNAs [14]. For instance, circ_101237 is reported to contribute to NSCLC development by upregulating MAPK1 via sponging miR-490-3p [15]. circ_0018818 is reported to aggravate NSCLC progression by increasing NID1 level via sequestering miR-767-3p [16]. We predicted the downstream miRNA/messenger RNA (mRNA) axis of circ_0002346 using bioinformatics databases, and its working mechanism was tested by rescue experiments.

In the current study, we first analyzed the characteristics and functions of circ_0002346 in NSCLC cells. The downstream miRNA/mRNA axis of circ_0002346 was predicted by bioinformatics analysis, and its working mechanism was tested by rescue experiments. Lastly, the *in vivo* role of circ_0002346 in the growth of xenograft tumors was evaluated using a xenograft tumor formation assay.

## 2. Materials and Methods

### 2.1. Clinical Specimens

NSCLC tissue specimens (*n* = 45) and paracancer normal tissue specimens (*n* = 45) were harvested from NSCLC patients at the Affiliated Xiangshan Hospital of Wenzhou Medical University. All patients were diagnosed by histopathological examination. The clinical tissue specimens were immediately frozen in liquid nitrogen and then stored at -80°C. A written informed consent form had been signed by all the participants, and this clinical study was authorized by the ethics committee of the Affiliated Xiangshan Hospital of Wenzhou Medical University.

### 2.2. Cell Lines

The human normal lung epithelial cell line (HBE) and two NSCLC cell lines (A549 and H1299) were purchased from the Type Culture Collection of the Chinese Academy of Sciences (Shanghai, China). All cell lines were cultured in Roswell Park Memorial Institute-1640 medium (RPMI-1640, Gibco, Carlsbad, CA, USA) with the addition of 10% fetal bovine serum (FBS, Gibco) and 1% penicillin/streptomycin (Sangon Biotech, Shanghai, China) at 37°C with 5% CO_2_.

### 2.3. Reverse Transcription-Quantitative Polymerase Chain Reaction (RT-qPCR)

RNA samples were isolated with the TRIzol Reagent (Ambion, Austin, TX, USA). For circRNA and mRNA, reverse transcription (RT) was conducted using a complementary DNA (cDNA) kit (Thermo Fisher Scientific, Waltham, MA, USA), and thermal cycling reaction was conducted using SYBR Green reagents (Invitrogen, Carlsbad, CA, USA). The fold changes were analyzed by the 2^−ΔΔCt^ method and normalized to *β*-actin. RT-qPCR of miR-582-3p was conducted using its specific Bulge-Loop™ miRNA RT-qPCR primer sets (RiboBio, Guangzhou, China), and the relative expression of miR-582-3p was analyzed by the 2^−ΔΔCt^ method and normalized to U6. Primer sequences are shown in Table 1.

### 2.4. RNase R Treatment

RNase R was purchased from Epicentre Technologies (Madison, WI, USA). RNA samples (4 *μ*g) were incubated with 12 U RNase R for 40 min at 37°C. The levels of circ_0002346 and its host gene cysteine rich transmembrane BMP regulator 1 (CRIM1) were examined by RT-qPCR.

### 2.5. Isolating RNAs in Nuclear and Cytoplasmic Fractions

The subcellular localization of circ_0002346 in NSCLC cells was analyzed by this assay. The nuclear and cytoplasmic fractions were isolated using the PARIS™ Kit (Invitrogen). U6 and 18S rRNA were regarded as positive references for the nucleus and cytoplasm, respectively.

### 2.6. Plasmids and Small RNAs

The circ_0002346 overexpression plasmid (circ_0002346), pLO-ciR vector (vector), circ_0002346 lentivirus vector (lenti-circ_0002346), negative control (lenti-NC), mimics of miR-582-3p (miR-582-3p), miR-NC, small interfering (si)RNA targeting STXBP6 (si-STXBP6), and si-NC were purchased from RiboBio. The Lipofectamine 3000 Reagent (Invitrogen) was utilized to transiently transfect plasmids and small RNAs into NSCLC cells.

### 2.7. 5-Ethynyl-2′-Deoxyuridine (EDU) Assay

Cell proliferation ability was assessed by an EDU assay. In brief, NSCLC cells were incubated with 30 *μ*M EDU (KeyGen Biotech, Jiangsu, China) for 2 h, and the cell nucleus was stained with 4,6-diamino-2-phenylindole (DAPI; Sigma-Aldrich, St. Louis, MO, USA). Cell fluorescence images were captured, and the EDU incorporation rate was evaluated.

### 2.8. Colony Formation Assay

Cell proliferation ability was evaluated by a colony formation assay. NSCLC cells were dispersed in culture medium at low density, and cells were seeded onto 12-well plates at 200 cells/well. Cells were incubated for 2 weeks to form visible colonies. The colonies were immobilized with 4% paraformaldehyde (Sigma-Aldrich) and stained with 0.1% crystal violet (Sigma-Aldrich). After washing twice and being air-dried, the number of colonies was analyzed.

### 2.9. Transwell Assays

A transwell migration or invasion assay was implemented to assess cell migration or invasion capacity using uncoated or Matrigel- (BD Biosciences, San Jose, CA, USA) covered transwell plates, respectively. NSCLC cells were dispersed in serum-free medium, and cells were then seeded onto the above chambers. The below chambers were added with 500 *μ*L medium plus 20% FBS (chemokines). After 24 h incubation, unmigrated or uninvaded NSCLC cells were scraped using a cotton swab, and cells that passed through the membrane were immobilized with 4% paraformaldehyde (Sigma-Aldrich) and stained with 0.5% crystal violet (Sigma-Aldrich). Cell number in five random fields was analyzed under a light microscope (Olympus, Osaka, Japan) at 100x.

### 2.10. Flow Cytometry

The apoptosis of NSCLC cells was analyzed by flow cytometry. The percentage of apoptosis cells (FITC^+^/PI^+/-^) was evaluated using an Annexin V-fluorescein isothiocyanate (FITC)/propidium iodide (PI) kit (Vazyme, Nanjing, China). After transfection for 72 h, NSCLC cells in 400 *μ*L binding buffer were simultaneously stained with 5 *μ*L Annexin V-FITC and 5 *μ*L PI in the dark. Apoptosis cells with FITC^+^/PI^+/-^ were captured by using a FACSCanto II flow cytometer (BD Biosciences), and the apoptosis rate was analyzed.

### 2.11. Analysis of Caspase3 Activity

The activity of caspase3 was analyzed using a caspase3 activity assay kit (Beyotime, Beijing, China). Briefly, cell extracts were incubated with the assay reagent and its specific substrate Ac-DEVD-pNA for 2 h. The absorbance was determined at the spectrum of 405 nm. The activity of caspase3 was assessed through drawing the standard curve.

### 2.12. Western Blot Assay

NSCLC cells were disrupted with a whole-cell lysis reagent (Thermo Fisher Scientific), and the concentrations of protein samples were analyzed using a BCA assay kit (Bio-Rad). Protein samples (30 *μ*g) were subjected to 10% separating gel and then transferred onto a polyvinylidene difluoride (PVDF) membrane (Millipore, Billerica, MA, USA). The membrane was sealed with 5% nonfat milk, incubated with primary antibodies against Bax (Bcl-2-associated X, apoptosis regulator, ab32503, Abcam, Cambridge, MA, USA), cleaved caspase3 (C-casp3, ab32042, Abcam), hexokinase 2 (HK2, ab209847, Abcam), pyruvate kinase M2 (PKM2, ab85555, Abcam), STXBP6 (HPA003552, Sigma-Aldrich), and *β*-actin (SAB3500350, Sigma-Aldrich), and then incubated with a secondary antibody (Sigma-Aldrich). Protein signals were visualized using Western Blotting Substrate (Thermo Fisher Scientific). The intensities of the protein bands were assessed by Image Lab analysis software (Bio-Rad).

### 2.13. Analysis of Glucose Uptake and Lactate Production

A fluorescence-based glucose/lactate assay kit (BioVision, Milpitas, California, USA) was used to analyze glucose uptake and lactate production according to the manufacturer's instructions.

### 2.14. Dual-Luciferase Reporter Assay

The downstream miRNA targets of circ_0002346 were predicted by the Circinteractome database (https://circinteractome.irp.nia.nih.gov), and miR-582-3p-mRNA interactions were predicted by the TargetScan database (http://www.targetscan.org).

The partial fragment of circ_0002346 or STXBP6 was subcloned to the luciferase vector pmirGLO (Promega, Madison, WI, USA), and the constructed plasmids were termed as circ_0002346 WT/MUT and STXBP6-3′ untranslated region (3′UTR) WT/MUT. After cotransfecting these luciferase plasmids and oligonucleotides into NSCLC cells for 24 h, the luciferase intensities were examined via a commercial dual-luciferase reporter assay system kit (Promega).

### 2.15. RNA Pull-Down Assay

miR-582-3p was biotinylated to construct Bio-miR-582-3p, and Bio-miR-NC was regarded as the control. Cell extracts (3 *μ*g) were incubated with 100 pmol Bio-miR-582-3p or Bio-miR-NC. The streptavidin agarose beads were then added to the tubes. RNA levels were examined by RT-qPCR.

### 2.16. *In Vivo* Xenograft Tumor Formation Assay

A549 cells stably expressing lenti-NC or lenti-circ_0002346 were subcutaneously inoculated into nude mice (5 mice/group). Tumor size was analyzed every 8 d for 40 d using the formula of tumor volume = length × width^2^ × 0.5. After subcutaneous injection for 40 d, the nude mice were sacrificed, and the subcutaneous xenograft tumors were removed and weighed. Tumors were then paraffin-embedded for an immunohistochemistry (IHC) assay. Male nude mice were purchased from Vital River Laboratory Animal Technology (Beijing, China) and were grown under pathogen-free conditions. All procedures in animal experiments have been approved by the Research Animal Care and Use Committee of the Affiliated Xiangshan Hospital of Wenzhou Medical University.

### 2.17. Statistical Analysis

Statistical analysis was conducted using GraphPad Prism 7.0 software (GraphPad, La Jolla, CA, USA), and the data were represented as mean ± standard deviation (SD). The differences were evaluated by Student's *t*-test and one-way analysis of variance (ANOVA) followed by Tukey's test. Differences were considered to be statistically significant at *P* < 0.05.

## 3. Results

### 3.1. circ_0002346 Expression Is Downregulated in NSCLC Tissues and Cell Lines

**Figure 1:**
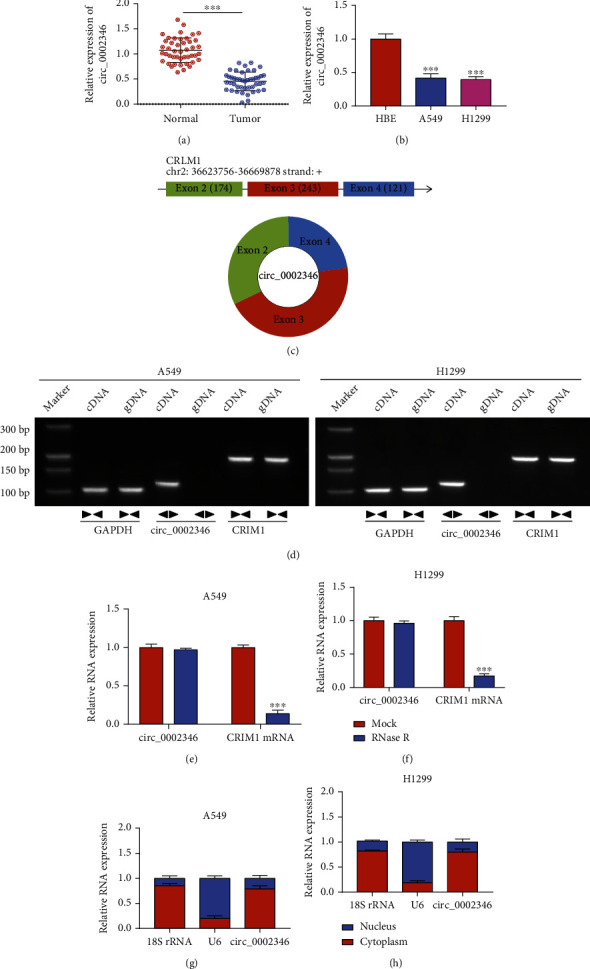
circ_0002346 expression is downregulated in NSCLC tissues and cell lines. (a) The expression level of circ_0002346 was examined in 45 pairs of NSCLC tissues and paracancer tissues by RT-qPCR. (b) RT-qPCR was conducted to measure the level of circ_0002346 in the NSCLC cell lines (A549 and H1299) and the HBE cell line. (c) A schematic diagram of the genomic location and structural composition of circ_0002346 was shown. (d) The existence of circ_0002346 and CRIM1 from cDNA and gDNA was verified by PCR using the divergent and convergent primers, respectively. (e, f) The RNase R resistance of circ_0002346 and its host gene CRIM1 mRNA was analyzed by RT-qPCR. (g, h) The subcellular localization of circ_0002346 was analyzed, and 18S rRNA and U6 served as cytoplasmic and nuclear markers, respectively. ^∗∗∗^*P* < 0.001.

We first analyzed the expression pattern of circ_0002346 in NSCLC tissues and cell lines. circ_0002346 expression was markedly reduced in NSCLC tissues and cell lines compared with adjacent healthy tissues and the HBE cell line (Figures 1(a) and 1(b)). circ_0002346 was generated from the back-splicing of exon 2 (174 nt), 3 (243 nt), and 4 (121 nt) of its host gene CRIM1 (Figure 1(c)). To confirm the circular structure of circ_0002346, divergent and convergent primers were used to amplify circ_0002346 and its linear counterpart CRIM1, respectively. The results revealed that circ_0002346 can be amplified in the cDNA group when using divergent primers, not in the gDNA group, suggesting that circ_0002346 was indeed a circular transcript (Figure 1(d)). circRNAs are resistant to RNase R due to their covalently closed continuous loop structure. circ_0002346 was resistant to RNase R degradation (Figures 1(e) and 1(f)), which further demonstrated that circ_0002346 was a circular transcript. The functions of circRNAs are closely associated with their subcellular localization. We found that circ_0002346 was majorly localized in the cytoplasmic fraction of NSCLC cells (Figures 1(g) and 1(h)), suggesting that circ_0002346 might regulate gene expression at the posttranscriptional level. Taken together, circ_0002346 was notably downregulated in NSCLC.

### 3.2. Overexpression of circ_0002346 Suppresses the Malignant Behaviors of NSCLC Cells

**Figure 2:**
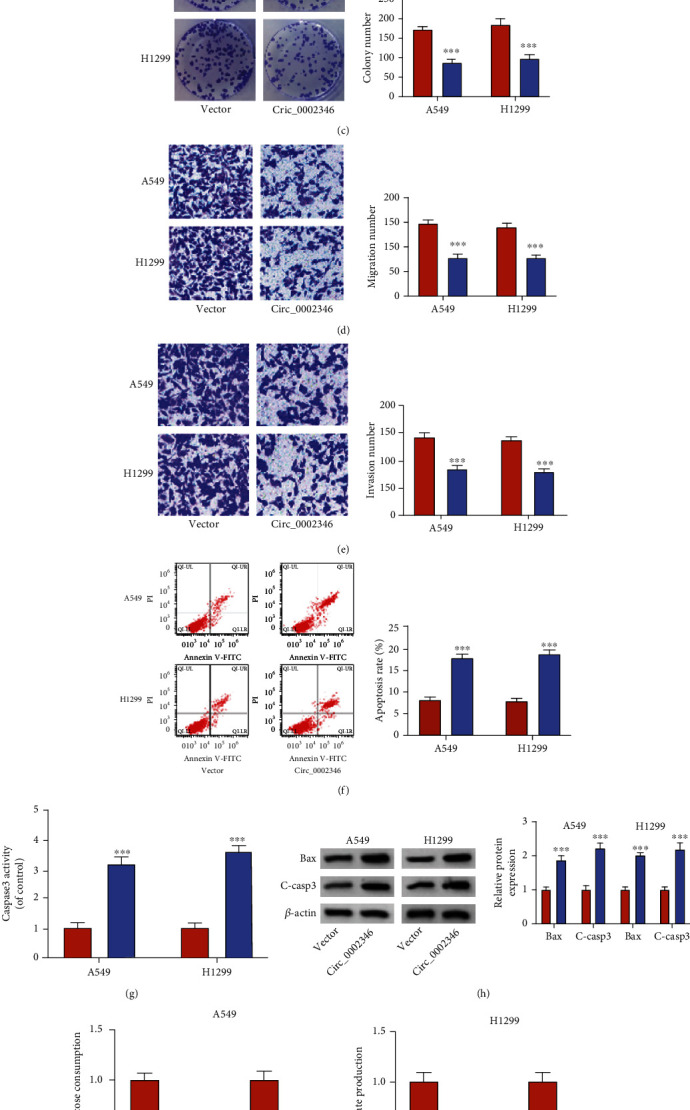
Overexpression of circ_0002346 suppresses the malignant behaviors of NSCLC cells. (a–k) NSCLC cells were transfected with vector or circ_0002346 plasmid. (a) The expression of circ_0002346 was determined by RT-qPCR. (b) An EDU assay was performed to assess the rate of DNA synthesis by measuring the incorporation of EDU. (c) A colony formation assay was conducted to measure the colony number to analyze cell proliferation ability. (d, e) Transwell assays were conducted to measure the numbers of migrated and invaded cells to analyze cell migration and invasion abilities. (f) Flow cytometry was conducted to analyze the cell apoptosis rate (the percentage of NSCLC cells with FITC^+^ and PI^+/-^). (g) The activity of caspase3 was analyzed using a caspase3 activity assay kit. (h) The expression of apoptosis-associated proteins (Bax and C-casp3) was measured in circ_0002346-overexpressed NSCLC cells by a Western blot assay. (i, j) The uptake of glucose and the production of lactate were determined using a fluorescence-based glucose/lactate assay kit. (k) A Western blot assay was conducted to measure the protein expression of HK2 and PKM2 in circ_0002346-overexpressed NSCLC cells. ^∗∗∗^*P* < 0.001.

To analyze the biological function of circ_0002346 in NSCLC cells, we performed gain-of-function experiments by transfecting circ_0002346 plasmid into NSCLC cells. circ_0002346 plasmid was effective in upregulating circ_0002346 level in NSCLC cells (Figure 2(a)). Cell proliferation ability was assessed by an EDU assay and a colony formation assay. circ_0002346 overexpression reduced the incorporation of EDU in NSCLC cells (Figure 2(b)), suggesting that circ_0002346 overexpression suppressed the proliferation of NSCLC cells. The number of colonies was decreased following the overexpression of circ_0002346 (Figure 2(c)), which further demonstrated that circ_0002346 overexpression restrained the proliferation ability of NSCLC cells. The numbers of migrated and invaded cells were both reduced after the overexpression of circ_00023646 (Figures 2(d) and 2(e)), demonstrating that circ_0002346 overexpression suppressed the migration and invasion of NSCLC cells. circ_0002346 overexpression markedly elevated the apoptosis rate of NSCLC cells (Figure 2(f)). Also, the activity of caspase3 was markedly increased following the overexpression of circ_0002346 (Figure 2(g)). circ_0002346 overexpression elevated the levels of proapoptotic proteins (Bax and C-casp3) (Figure 2(h)). These results suggested that circ_0002346 overexpression induced the apoptosis of NSCLC cells. circ_0002346 overexpression reduced the uptake of glucose and the production of lactate (Figures 2(i) and 2(j)). Moreover, circ_0002346 accumulation reduced the levels of two glycolysis-associated rate-limiting enzymes (HK2 and PKM2) in NSCLC cells (Figure 2(k)). These results suggested that circ_0002346 overexpression restrained cell glycolytic metabolism. Taken together, circ_0002346 played a tumor suppressor role by inhibiting the malignant behaviors of NSCLC cells.

### 3.3. circ_0002346 Directly Binds to miR-582-3p

**Figure 3:**
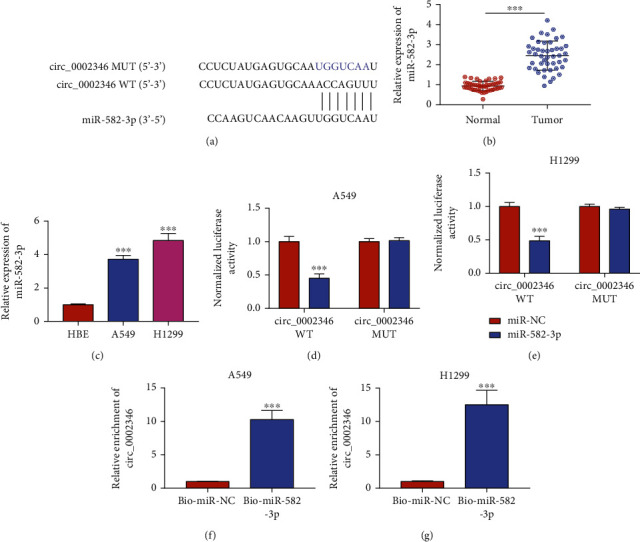
circ_0002346 directly binds to miR-582-3p. (a) The putative binding sites between circ_0002346 and miR-582-3p predicted by Circinteractome are shown. (b) The expression of miR-582-3p in 45 pairs of NSCLC tissues and paracancer tissues was determined by RT-qPCR. (c) The level of miR-582-3p was examined in the NSCLC cell lines and the HBE cell line by RT-qPCR. (d, e) The target relationship between circ_0002346 and miR-582-3p was verified by a dual-luciferase reporter assay. (f, g) An RNA pull-down assay was performed to confirm the interaction between circ_0002346 and miR-582-3p. ^∗∗∗^*P* < 0.001.

Accumulating evidence has demonstrated that circRNAs can function as oncogenes or tumor suppressors by sponging miRNAs [6, 17]. Through bioinformatics analysis using the Circinteractome database, miR-582-3p was predicted as a target of circ_0002346. The putative binding sites between circ_0002346 and miR-582-3p are shown in Figure 3(a). miR-582-3p expression was notably upregulated in NSCLC tissues and cell lines relative to that in adjacent normal tissues and the HBE cell line (Figures 3(b) and 3(c)). Subsequently, a dual-luciferase reporter assay and an RNA pull-down assay were conducted to confirm the interaction between circ_0002346 and miR-582-3p. The dual-luciferase reporter assay showed that miR-582-3p overexpression can markedly reduce the luciferase activity driven by the wild-type plasmid (circ_0002346 WT) instead of the mutant plasmid (circ_0002346 MUT) (Figures 3(d) and 3(e)), suggesting that circ_0002346 directly interacted with miR-582-3p via the predicted sites. circ_0002346 was pulled down when using a Bio-miR-582-3p probe compared with that in the Bio-miR-NC group (Figures 3(f) and 3(g)), demonstrating the binding relationship between circ_0002346 and miR-582-3p. Taken together, miR-582-3p was a direct target of circ_0002346 in NSCLC cells.

### 3.4. circ_0002346 Overexpression-Induced Antitumor Impacts Are Partly Reversed by the Addition of miR-582-3p Mimics in NSCLC Cells

**Figure 4:**
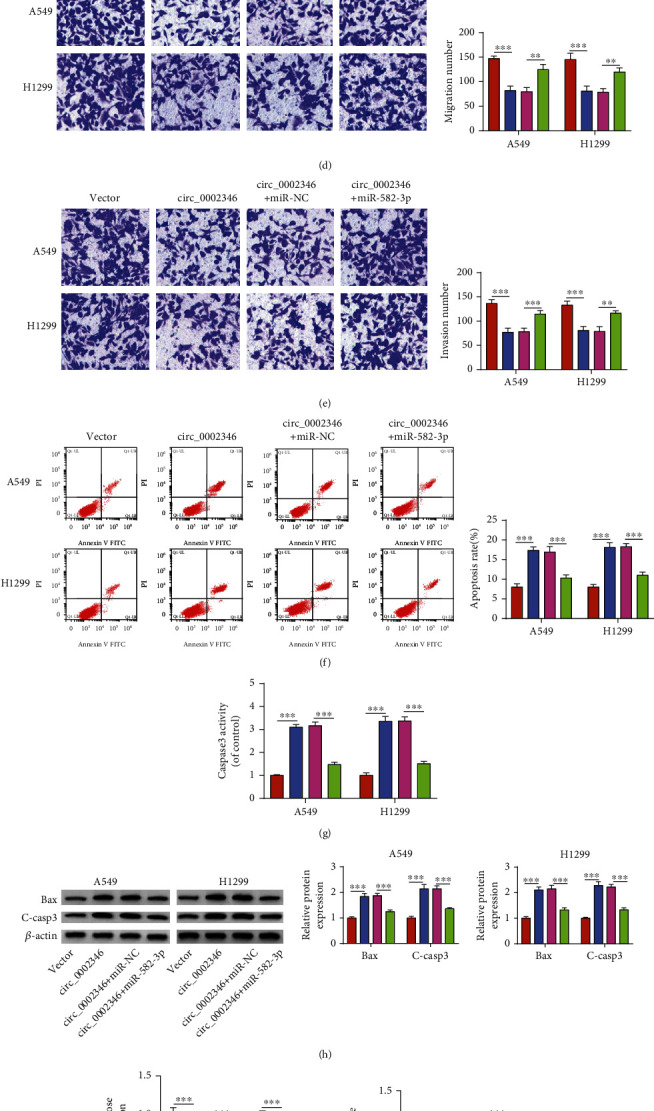
circ_0002346 overexpression-induced antitumor impacts are partly reversed by the addition of miR-582-3p mimics in NSCLC cells. (a) The transfection efficiency of miR-582-3p mimics in NSCLC cells was analyzed by RT-qPCR. (b–k) NSCLC cells were transfected with circ_0002346 alone or together with miR-582-3p mimics. (b, c) Cell proliferation ability was measured by an EDU assay and a colony formation assay. (d, e) Cell migration and invasion abilities were analyzed by transwell assays. (f) The apoptosis rate (FITC^+^ and PI^+/-^) of NSCLC cells was assessed by flow cytometry. (g) Cell apoptosis was analyzed by measuring caspase3 activity using a caspase3 activity assay kit. (h) The protein expression of Bax and C-casp3 was detected by a Western blot assay. (i, j) The consumption of glucose and the level of lactate were analyzed using a fluorescence-based glucose/lactate assay kit. (k) The protein levels of HK2 and PKM2 were detected by a Western blot assay. ^∗∗^*P* < 0.01; ^∗∗∗^*P* < 0.001.

An RT-qPCR assay confirmed the high transfection efficiency of miR-582-3p mimics in NSCLC cells (Figure 4(a)). We wondered whether circ_0002346 overexpression-mediated antitumor effects were partly based on its sponge activity for miR-582-3p, and rescue experiments were performed by transfecting NSCLC cells with circ_0002346 alone or together with miR-582-3p mimics. An EDU assay and a colony formation assay together demonstrated that the addition of miR-582-3p mimics partly rescued the proliferation ability of NSCLC cells (Figures 4(b) and 4(c)). The introduction of miR-582-3p also largely recovered the migration and invasion abilities of NSCLC cells (Figures 4(d) and 4(e)). miR-582-3p overexpression attenuated circ_0002346 overexpression-induced apoptosis in NSCLC cells (Figures 4(f)–4(h)), evidenced by the reduced apoptosis rate, the activity of caspase3, and the protein levels of Bax and C-casp3. circ_0002346 overexpression restrained cell glycolytic metabolism, and the introduction of miR-582-3p mimics largely rescued the glycolytic rate of NSCLC cells (Figures 4(i)–4(k)), verified by the increased rates of glucose consumption and lactate production and the protein levels of HK2 and PKM2. Taken together, circ_0002346 overexpression suppressed NSCLC progression partly by downregulating miR-582-3p.

### 3.5. STXBP6 Is a Target of miR-582-3p

**Figure 5:**
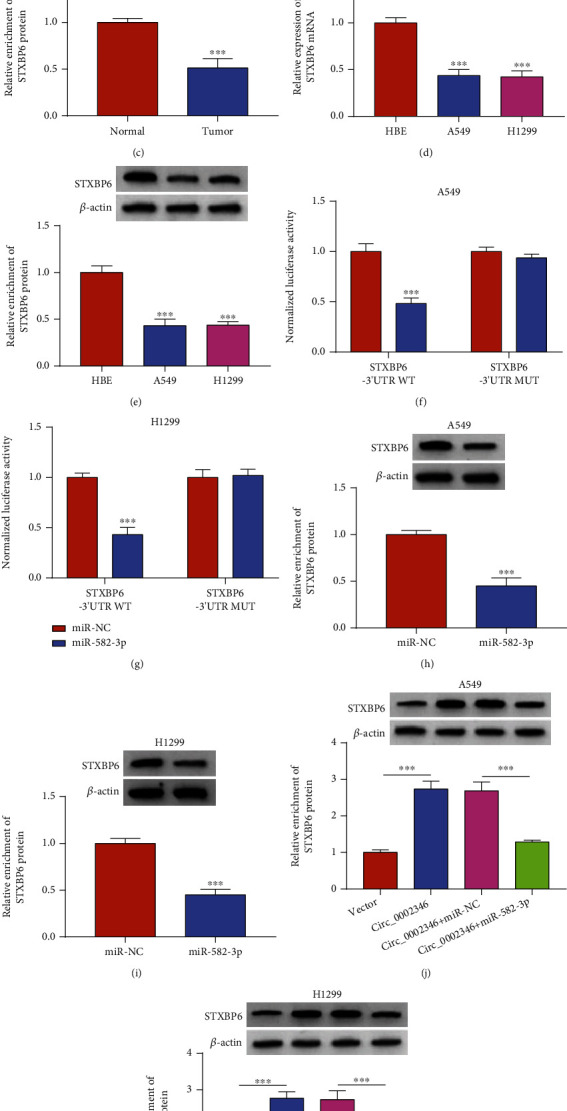
STXBP6 is a target of miR-582-3p. (a) The putative binding sequence between miR-582-3p and STXBP6 was predicted by the TargetScan database. (b, c) The mRNA and protein expression of STXBP6 was determined in NSCLC tissues and adjacent normal tissues by RT-qPCR and a Western blot assay. (d, e) RT-qPCR and a Western blot assay were conducted to analyze the mRNA and protein expression of STXBP6 in the NSCLC cell lines and the HBE cell line. (f, g) A dual-luciferase reporter assay was performed to verify the interaction between miR-582-3p and STXBP6. (h, i) The protein expression of STXBP6 was measured in NSCLC cells transfected with miR-NC or miR-582-3p by a Western blot assay. (j, k) NSCLC cells were transfected with circ_0002346 alone or together with miR-582-3p, and a Western blot assay was conducted to detect the protein level of STXBP6 in NSCLC cells. ^∗∗∗^*P* < 0.001.

miRNAs can bind to the 3′UTR of mRNAs to induce the degradation or translational repression of target mRNAs [18]. Bioinformatics analysis using the TargetScan database showed that the 3′UTR of STXBP6 harbored the complementary sites with miR-582-3p (Figure 5(a)). The mRNA and protein expression of STXBP6 was significantly reduced in NSCLC tissues compared with normal tissues (Figures 5(b) and 5(c)). Compared with the HBE cell line, the mRNA and protein levels of STXBP6 were reduced in NSCLC cell lines (Figures 5(d) and 5(e)). The luciferase activity of the wild-type plasmid (STXBP6-3′UTR WT) was markedly reduced by the overexpression of miR-582-3p (Figures 5(f) and 5(g)). However, the luciferase activity of the mutant plasmid (STXBP6-3′UTR MUT) remained unchanged upon the transfection of miR-NC or miR-582-3p (Figures 5(f) and 5(g)), suggesting that miR-5582-3p interacted with the 3′UTR of STXBP6 via the predicted sites. The overexpression of miR-582-3p significantly reduced STXBP6 protein level in NSCLC cells (Figures 5(h) and 5(i)). circ_0002346 overexpression notably increased the protein expression of STXBP6, and the introduction of miR-582-3p mimics led to a prominent reduction in the protein expression of STXBP6 in NSCLC cells (Figures 5(j) and 5(k)). Taken together, these results indicated that STXBP6 was a direct target of miR-582-3p, and it was regulated by the circ_0002346/miR-582-3p axis.

### 3.6. STXBP6 Silencing Partly Offsets circ_0002346 Overexpression-Induced Effects in NSCLC Cells

**Figure 6:**
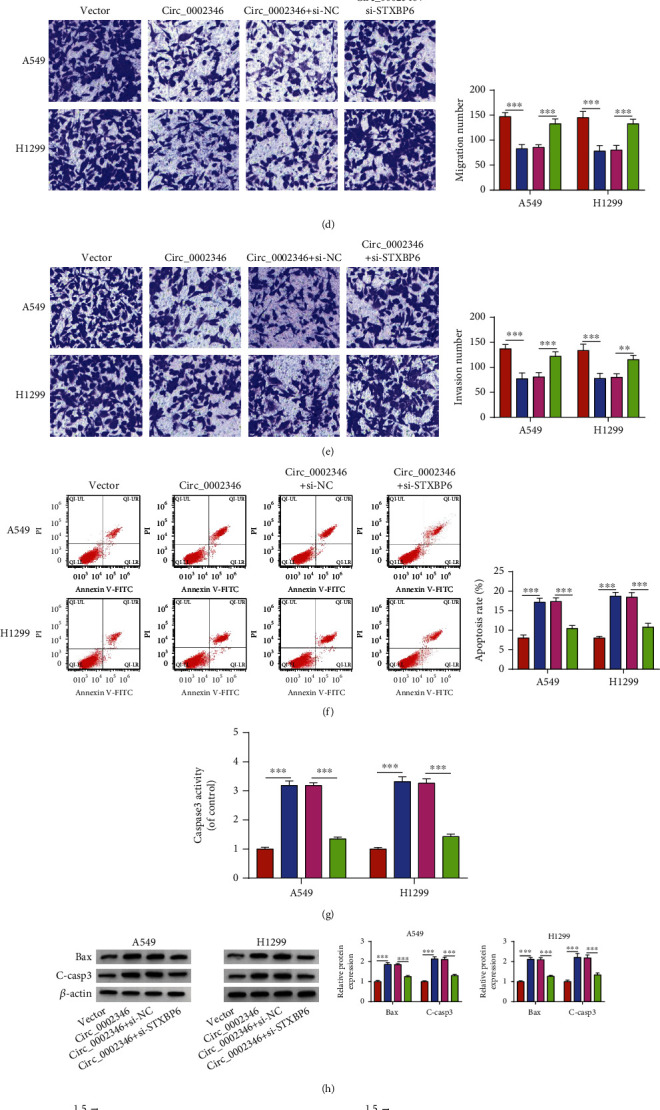
STXBP6 silencing partly offsets circ_0002346 overexpression-induced effects in NSCLC cells. (a) A Western blot assay was conducted to measure the protein expression of STXBP6 in NSCLC cells transfected with si-NC or si-STXBP6. (b–k) A549 and H1299 cells were transfected with circ_0002346 alone or together with si-STXBP6. (b and c) An EDU assay and a colony formation assay were performed to measure cell proliferation ability. (d, e) Transwell assays were conducted to analyze cell migration and invasion abilities. (f) The apoptosis rate of NSCLC cells (FITC^+^ and PI^+/-^) was analyzed by flow cytometry. (g) The activity of caspase3 was analyzed using a caspase3 activity assay kit. (h) The protein levels of Bax and C-casp3 were measured by a Western blot assay. (i, j) The uptake of glucose and the production of lactate were measured using a fluorescence-based glucose/lactate assay kit. (k) The protein expression of HK2 and PKM2 was examined in NSCLC cells by a Western blot assay. ^∗∗^*P* < 0.01; ^∗∗∗^*P* < 0.001.

RT-qPCR showed that the transfection efficiency of si-STXBP6 was high in NSCLC cells (Figure 6(a)). To explore the functional association between circ_0002346 and STXBP6, we conducted rescue experiments by transfecting NSCLC cells with the circ_0002346 plasmid alone or together with si-STXBP6. The addition of si-STXBP6 largely rescued the proliferation ability of NSCLC cells (Figures 6(b) and 6(c)), evidenced by the increased ratio of EDU incorporation and colony number. The introduction of si-STXBP6 also largely rescued the migration and invasion abilities of NSCLC cells (Figures 6(d) and 6(e)). STXBP6 silencing attenuated circ_0002346 overexpression-induced apoptosis in NSCLC cells (Figure 6(f)). Furthermore, the introduction of si-STXBP6 reduced the activity of caspase3 and decreased the protein levels of Bax and C-casp3 in circ_0002346-overexpressed NSCLC cells (Figures 6(g) and 6(h)). circ_0002346 overexpression-induced suppressive effects on the glucose consumption and lactate production were largely reversed by the addition of si-STXBP6 (Figures 6(i) and 6(j)). The addition of si-STXBP6 largely rescued the protein levels of HK2 and PKM2 in circ_0002346-overexpressed NSCLC cells (Figure 6(k)). Taken together, circ_0002346 overexpression suppressed the malignant behaviors of NSCLC cells partly by upregulating STXBP6.

### 3.7. circ_0002346 Overexpression Suppresses Xenograft Tumor Growth *In Vivo*

**Figure 7:**
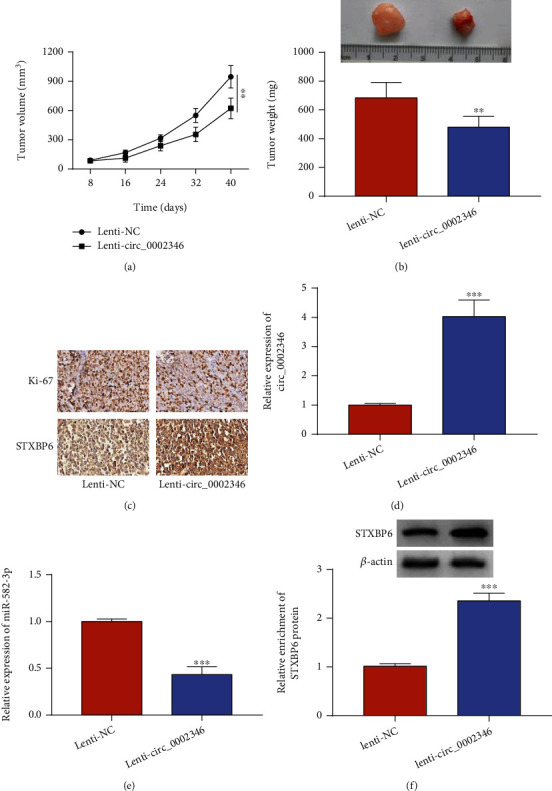
circ_0002346 overexpression suppresses xenograft tumor growth *in vivo*. (a) Tumor volume was monitored every 8 d for 40 d as length × width^2^ × 0.5. (b) Tumor weight was recorded after inoculation for 40 d. (c) An IHC assay was conducted to analyze the protein levels of Ki-67 and STXBP6 in tumor tissues. (d, e) RT-qPCR was conducted to measure the levels of circ_0002346 and miR-582-3p in tumor tissues. (f) A Western blot assay was performed to detect the protein level of STXBP6 in tumor tissues. ^∗∗^*P* < 0.01; ^∗∗∗^*P* < 0.001.

Considering the tumor suppressor role of circ_0002346 *in vitro*, we then analyzed the biological role of circ_0002346 on tumor growth using a xenograft tumor formation assay *in vivo*. The results indicated that the average volume of xenograft tumors in the lenti-circ_0002346 group was notably smaller than that in the lenti-NC group (Figure 7(a)). Meanwhile, tumor weight was also reduced in the lenti-circ_0002346 group relative to the lenti-NC group (Figure 7(b)). The results of an IHC assay suggested that the staining intensity of Ki-67 was notably decreased in circ_0002346-overexpressed tumor tissue compared with the lenti-NC group (Figure 7(c)). In addition, we found that the staining intensity of STXBP6 was markedly increased in circ_0002346-overexpressed tumor tissue in comparison with the lenti-NC group (Figure 7(c)). Moreover, we measured the expression of the circ_0002346/miR-582-3p/STXBP6 axis in tumor tissues. The expression of circ_0002346 and STXBP6 was significantly upregulated in circ_0002346-overexpressed tissues, and miR-582-3p level was reduced in the circ_0002346-overexpressed group (Figures 7(d)–7(f)). Taken together, circ_0002346 overexpression restrained tumor growth *in vivo*.

## 4. Discussion

Owing to the rapid progression of sequencing technology and genomic research, more and more circRNAs have been identified and studied [19, 20]. Unlike linear noncoding RNAs, such as miRNAs, circRNAs are stable circular noncoding RNAs that are resistant to exonuclease digestion [21]. Previous evidence has demonstrated that circRNAs are implicated in NSCLC progression. For example, circ_0000376 is reported to contribute to the proliferation, motility, and chemoresistance of NSCLC cells by sequestering miR-384 [22]. circ_0002483 is reported to increase the Taxol sensitivity of NSCLC cells by absorbing miR-182-5p [23]. Wang et al. found that circ_0002346 expression is decreased in lung adenocarcinoma, and it restrains the invasion and motility of lung adenocarcinoma cells by targeting miR-182 and miR-93 [24]. Consistent with a former study, we found that circ_0002346 level was markedly reduced in NSCLC tissue specimens and cell lines. circ_0002346 overexpression restrained the proliferation, migration, invasion, and glycolysis and facilitated the apoptosis of NSCLC cells.

Accumulating articles pointed out that circRNAs can regulate gene expression and multiple cellular biological processes by absorbing miRNAs [17, 25]. Moreover, one circRNA can absorb many diverse miRNAs. For instance, circ_0008532 is reported to contribute to bladder cancer development by upregulating MTGR1 via sponging miR-155-5p and miR-330-5p [26]. circ-PRMT5 is reported to aggravate gastric cancer development by absorbing miR-145 and miR-1304 [27]. We found that miR-582-3p was a novel target of circ_0002346 in addition to miR-182 and miR-93 [24]. miR-582-3p level was elevated in NSCLC. Fang et al. reported that miR-582-3p maintains the malignant properties of lung cancer stem cells by activating the Wnt/*β*-catenin pathway [28]. Wang et al. found that miR-582-3p level is elevated in hypoxic NSCLC cell-derived exosomes, and it can be transmitted to normoxic NSCLC cells via exosomes, thereby promoting the malignant properties of normoxic NSCLC cells [29]. These studies demonstrated the oncogenic role of miR-582-3p in NSCLC cells. Consistently, circ_0002346 overexpression-induced inhibitory effects on the malignant properties of NSCLC cells were partly attenuated by the addition of miR-582-3p mimics, suggesting that circ_0002346 suppressed NSCLC progression partly by downregulating the oncogenic molecule miR-582-3p.

miRNAs can bind to the 3′UTR of mRNAs to induce the degradation or translational repression of target mRNAs [18]. To further explore the regulatory mechanism of the circ_0002346/miR-582-3p axis in NSCLC progression, bioinformatics analysis was performed and we found that STXBP6 was a possible target of miR-582-3p. The binding relationship between miR-582-3p and STXBP6 was then validated by a dual-luciferase reporter assay. STXBP6 expression was decreased in NSCLC, and it was reversely regulated by miR-582-3p in NSCLC cells. The aberrant expression of STXBP6 is reported to be associated with multiple human diseases, including diabetes [30], autism [31], and cancers [32, 33]. A previous study showed that STXBP6 overexpression inhibits the proliferation and motility and facilitates the apoptosis of lung adenocarcinoma cells, and low level of STXBP6 is associated with dismal prognosis of lung adenocarcinoma patients [34]. Here, rescue experiments revealed that STXBP6 silencing partly reversed circ_0002346 overexpression-induced antitumor effects in NSCLC cells, demonstrating that circ_0002346 overexpression restrained NSCLC progression partly by upregulating STXBP6. circ_0002346 can positively regulate STXBP6 expression by sponging miR-582-3p in NSCLC cells.

Given that circ_0002346 played a tumor suppressor role *in vitro*, we further analyzed whether circ_0002346 overexpression suppressed tumor growth *in vivo*. The results showed that circ_0002346 overexpression markedly restrained xenograft tumor growth *in vivo*, and its tumor suppressor role was at least partly based on the regulation of the miR-582-3p/STXBP6 axis.

In conclusion, our findings demonstrated that circ_0002346 suppressed NSCLC progression partly by targeting the miR-582-3p/STXBP6 axis *in vitro* and *in vivo*, which provided novel potential targets for NSCLC treatment.

## Figures and Tables

**Table 1:** Primer sequences.

Gene	Sequence (5′-3′)
circ_0002346	Forward: CCCGGACAGCTATGAAACTC Reverse: GCAGCCAGCAATAAGGTTTT
CRIM1	Forward: ACGCCGCGGATCTTGTG Reverse: GCTCCGGTTCAACCCAAACT
miR-582-3p	Forward: GCCGAGTAACTGGTTGAACA Reverse: CTCAACTGGTGTCGTGGA
STXBP6	Forward: TGGAGTTGCCGGGAGTTTC Reverse: AACTCCACACACAAAGCACC
*β*-Actin	Forward: CTCGCCTTTGCCGATCC Reverse: TCTCCATGTCGTCCCAGTTG
U6	Forward: CTCGCTTCGGCAGCACA Reverse: AACGCTTCACGAATTTGCGT

## Data Availability

No data were used to support this study.
